# Effect of polycan, a β-glucan originating from *Aureobasidium,* on a high-fat diet-induced hyperlipemic hamster model

**DOI:** 10.3892/etm.2015.2238

**Published:** 2015-01-29

**Authors:** MEE-KYOUNG LIM, SAE-KWANG KU, JAE-SUK CHOI, JOO-WAN KIM

**Affiliations:** 1 Department of Food Science and Nutrition, School of Human Ecology, Kyungpook National University, Daegu 702-701, Republic of Korea; 2 Department of Anatomy and Histology, College of Oriental Medicine, Daegu Haany University, Gyeongsan 712-715, Republic of Korea; 3 Department of Bio-Food Materials, Silla University, Busan 617-736, Republic of Korea; 4 Glucan Corporation, Marine Bio-Industry Development Center, Busan 619-912, Republic of Korea

**Keywords:** β-glucan, high-fat diet, hyperlipemia, atherosclerosis, hamster

## Abstract

The aim of the present study was to analyze the effect of polycan, a β-glucan originating from *Aureobasidium*, on high-fat diet (HFD)-induced hyperlipemia and hepatic damage. A total of 30 hamsters were divided into 6 groups based on their body weight following acclimatization: control, sham, simvastatin (SIMVA) and 3 Polycan groups. In the polycan groups, Polycan, at three concentrations (31.25, 62.5 and 125 mg/kg), was administered orally once a day for 56 days, in addition to the HFD. On the day of sacrifice, changes in the body weight, food consumption, liver weight and serum levels of aspartate aminotransferase (AST), alanine aminotransferase (ALT), low-density lipoprotein (LDL), high-density lipoprotein (HDL), triglyceride and total cholesterol (T-CHOL) were observed, as well as changes to the liver and aorta (thoracic and abdominal) histopathology and histomorphometry. The results from the polycan groups were compared with a SIMVA 10 mg/kg oral treatment group, in addition to the sham and vehicle control groups. After the HFD-induced hyperlipidemic hamsters were administered Polycan, there was no significant change in their body weight and food consumption when compared with the hamsters in the vehicle control group. However, the serum levels of AST, ALT, triglyceride, T-CHOL and LDL were significantly reduced in a dose-dependent manner when compared with the vehicle control group (P<0.05). Furthermore, the levels of liver steatosis and arteriosclerosis in the abdominal and thoracic aorta were significantly decreased in a dose-dependent manner (P<0.01). In the SIMVA-treated group, body weight (P<0.05), the serum level of lipids (triglyceride, T-CHOL and LDL; P<0.01) and the level of arteriosclerosis (P<0.01) were significantly reduced when compared with the vehicle control group. However, liver weight and the serum levels of AST, ALT, and liver steatosis increased when compared with the vehicle control group. Based on these results, it was concluded that polycan exerts a favorable effect in decreasing HFD-induced hyperlipemia and associated atherosclerosis, with relatively good protective effects on liver damage.

## Introduction

High blood cholesterol levels are considered to be one of the most significant risk factors contributing to the severity and prevalence of coronary heart disease (1,2). Generally, a diagnosis of hyperlipidemia is confirmed in individuals with blood cholesterol levels of >200 mg/dl or blood triglyceride levels of >180 mg/dl. Furthermore, hyperlipemia can be induced by the secondary effects of diabetes (3), and liver damage is often induced under conditions of hyperlipemia, as shown by a marked increase in the serum levels of aspartate aminotransferase (AST) and alanine aminotransferase (ALT) (4).

In humans, atherosclerosis is a focal disease that has been shown to evolve in a distinct pattern, resulting in atheroma formation and vessel obstruction (5,6). Considering the complexity of lesion development, the sequence of events and underlying mechanisms that occur are difficult to analyze in humans. One of the main challenges is the development of a suitable animal model that closely imitates the human disease. Although there is no perfect animal model, animal models can be useful to sequentially investigate the pathological alterations, from the initiation of the disease to the final stages of atherosclerotic plaque development. Irrespective of the species, the induction of vascular lesions is dependent upon hypercholesterolemia. The elevation of plasma cholesterol levels can be induced by a variety of methods, including dietary supplementation, hepatic overproduction of lipoproteins or the genetic mutation of receptors and/or receptor ligands that are responsible for lipoprotein clearance. Previously, the golden Syrian hamster has been successfully used to investigate vascular changes that occur during atherogenesis (7). When compared with other animal models, the hamster has a number of advantages. Firstly, similarly to humans, the main plasma cholesterol carrier is low-density lipoprotein (LDL), and lipoprotein metabolism exhibits similarities to that of humans (8). Furthermore, the hamster LDL receptor gene has been isolated and characterized (9), and has been demonstrated to have a number of similarities to the human gene. In addition, atherosclerotic plaques develop with predilection in the aortic arch, the aortic aspect of the sigmoid valves and the coronary arteries, all lesion-prone areas, which allows reliable assessment of the atherosclerotic process.

HMG-CoA reductase inhibitors have been used as treatment for hyperlipemia (10), and simvastatin (SIMVA) is one of the most prevalently used HMG-CoA reductase inhibitors (11). Therefore, SIMVA was used in the present study as a reference drug. β-glucan is a fiber-type complex sugar (polysaccharide) derived from the cell wall of baker’s yeast, oat and barley fiber, as well as numerous medicinal mushrooms, including maitake. The two primary uses of β-glucan are the enhancement of the immune system (12,13) and to decrease the levels of blood cholesterol (14,15). Previous clinical and animal studies have used concentrated β-glucan preparations from oats and barley and have demonstrated strong hypolipemic and associated anti-atherosclerosis effects on hypercholesterolemic hamsters (16,17). Although certain studies have demonstrated evidence of the direct effects of β-glucan on hepatopathies (18,19), the direct effects of β-glucan on hyperlipemic liver damage are seldom. In addition, the effects of the β-glucan originating from *Aureobasidium* on hypolipemia and associated anti-atherosclerosis have not yet been reported. The β-glucan used in the present study was extracted from *Aureobasidium pullulans* SM-2001 (primarily β-1,3/1,6-glucans), which is a UV-induced mutant of *A. pullulans*. Thus, the β-glucan is known to demonstrate somewhat different characteristics from β-glucan derived from other origins (20).

In the present study, the hypolipemic and associated anti-atherosclerosis effects of polycan (β-glucan; Glucan Corporation, Busan, Korea), originating from *Aureobasidium*, were observed on a high-fat diet (HFD)-induced hamster model of hyperlipemia, with possible effects on liver damage also assessed. The effects were evaluated based on the serum levels of AST, ALT, LDL, high-density lipoprotein (HDL), total cholesterol (T-CHOL) and triglyceride, with changes in the histology and histomorphometry of the liver and aorta (thoracic and abdominal) also analyzed (5,21,22).

## Materials and methods

### Animals

In total, 30 male hamsters (age, 7 weeks; Samtako Bio Korea Co., Ltd., Osan, Korea) were used in the study following acclimatization for 19 days. The 30 hamsters were grouped into 6 groups. This was carried out by arranging the hamsters in order of weight, the heaviest 6 hamsters were randomly assigned to each of the 6 groups. The subsequent 6 heaviest hamsters were then randomly assigned to each of the 6 groups; this division process was continued until the 30 hamsters had been randomly assigned to the 6 groups. The animals were allocated five per polycarbonate cage in a temperature (20–25°C) and humidity (40–45%) controlled room. A 12-h light/dark cycle was applied, and food and water were supplied *ad libitum*. The present study was approved by the ethics committee of Daegu Haany University (Gyeongsan, Korea)

### Preparations and administration of drugs

Polycan (β-glucan extract from *A. pullulans*) (20) and SIMVA (Sigma-Aldrich, St. Louis, MO, USA) were used as test articles in the study. Polycan was stored in a refrigerator at 4°C for protection against light and degeneration. Polycan was diluted in distilled water and dosed by oral gavage using a sonde attached to a 1-ml syringe, which contained the test article at a dose of 31.25, 62.5 or 125 mg/kg in distilled water, once a day for 56 days. In addition, SIMVA was orally administered at 10 mg/kg using distilled water as a vehicle.

### Hyperlipemia induction

To induce hyperlipemia, the animals were supplied with free access to a HFD (Dyets, Inc., Bethlehem, PA, USA), containing 1% cholesterol and 0.25% sodium cholate for 8 weeks, after a 19-day acclimatization period (23–26). The constituents of the HFD are listed in Table I. In the sham group, a normal pellet diet (Samyang Foods Co., Ltd., Wonju, Korea) was supplied *ad libitum* for the same time period.

### Body weight change

Changes in the body weight of the hamsters were calculated one day prior to drug administration (day −1), on the day of drug administration (day 0) and at days 1, 7, 14, 21, 28, 35, 42, 49, 55 and 56 after administration of the test article and HFD supply. On the first day of test article administration and at sacrifice, all the experimental animals had fasted overnight (water was not restricted) to reduce the erratum arousal from feeding. In addition, the gain in body weight (body weight on day 56 - body weight on day 0) was calculated.

### Measurement of food consumption

Food consumption was calculated weekly during the experimental period. The amount of food was measured prior to supplying each cage, and the subsequent remnants were measured the next day to calculate the difference, which was regarded as the daily food consumption per group [mean food consumption (g/day per animal) = daily food consumption per group/number of animals in each group].

### Liver weight changes

At sacrifice, the weight of the liver was calculated. In order to reduce the erratum originating from individual body weight differences, the relative weight (%) was calculated by dividing the absolute weight by the body weight at sacrifice and multiplying by 100.

### Serum biochemistry

At sacrifice, a 2-ml sample of venous blood was collected from the vena cava under anesthesia. All blood samples were centrifuged at 600 × g for 10 min at room temperature using a clotting activated serum tube. Serum AST and ALT levels were detected with an automated blood analyzer (Toshiba 200FR; Toshiba Medical Systems Corporation, Otawara-shi, Japan) and measured in IU/l, using kinetic UV methods. Briefly, when the AST or ALT enzymes reacted with the substrate, the NADH was oxidized to NAD. By measuring the reduction of the UV absorbance of NADH, the levels of AST or ALT were determined using the automatic blood analyzer. Serum LDL, HDL, triglyceride and T-CHOL levels were detected with an automated blood analyzer (AU400; Olympus Corporation, Tokyo, Japan), in mg/dl units, using an enzyme assay.

### Histopathological procedures

After measuring the liver weight, the liver and thoracic and abdominal aorta were sampled. The sampled organs were fixed in 10% neutral-buffered formalin. Following paraffin embedding, 3–4-μm sections were prepared. Representative sections were stained with hematoxylin and eosin for light microscopy examination, following which the histological profiles of the individual livers and aortas were observed (Eclipse 80i; Nikon Corporation, Tokyo, Japan).

### Histomorphometry

The percentage of degenerative regions (fatty changes) in the hepatic parenchyma was calculated as the percentage change between one randomly selected field of the liver (%/200 μm^2^ hepatic parenchyma) using an automated image analysis system (analySIS Image Processing; SiS Sensoren Instrumente Systeme GmbH, Schwentinental, Germany). The percentage of atherosclerotic plaque regions on the aorta surface was calculated as the percentage in a 1-mm section of the aorta surface (%/1 mm aorta surface), using an automated image analysis system (DMI-300; DMI, Seoul, South Korea).

### Statistical analysis

All data are expressed as the mean ± standard deviation. Statistical analyses were conducted with a Mann-Whitney-Wilcoxon test (MW test), using SPSS software for Windows (Release 14K; SPSS, Inc., Chicago, IL, USA). The inhibition rate compared with the vehicle control group was calculated to aid understanding of the efficacy of test materials on the differences between the sham and vehicle control [percentage change vs. sham (%) = (data of vehicle control - data of sham)/data of sham ×100] and vehicle control and test groups [percentage change vs. vehicle control (%) = (data of test group - data of vehicle control)/data of vehicle control ×100]. P<0.05 was considered to indicate a statistically significant difference.

## Results

### Changes to the body weight

A statistically significant (P<0.05) increase in body weight was detected between the hamsters that were supplied with the HFD for 49 days compared with those in the sham group that were fed a normal diet. In addition, the body weight gain throughout the whole experimental period significantly (P<0.05) increased. In the SIMVA group, a non-significant decrease in the body weight was detected from day 42 after administration, with a statistically significant (P<0.05) decrease observed in the body weight gain when compared with the vehicle control. However, no statistically significant differences were detected in any of the polycan groups (Table II) when compared with the sham or vehicle control groups.

In the vehicle control group, the body weight gain throughout the experimental period was shown to increase by 66.71% when compared with the sham group. In the SIMVA, polycan 31.25, 62.5 and 125 mg/kg groups, the changes in the body weight gain over the experimental period were found to be −54.06, −19.83, −9.46, and −8.88% when compared with vehicle control group, respectively.

### Food consumption

Statistically significant (P<0.01 or P<0.05) decreases were detected in food consumption when comparing all the HFD supplied groups, including the vehicle control, with the sham group who were fed a normal diet. However, no significant changes were identified in food consumption when comparing the treatment groups with the vehicle control (Table III).

The mean daily food consumption per animal was detected as 7.18±1.11, 5.30±0.83, 5.35±6.16, 5.13±0.61, 5.22±0.64 and 5.41±0.62 g/day per animal in the sham, vehicle control, SIMVA, polycan 31.25, 62.5 and 125 mg/kg groups, respectively.

### Changes in the liver weight

Statistically significant (P<0.01) increases were identified in the absolute and relative liver weights when comparing the vehicle control and the normal diet supplied sham group. In the SIMVA group, a statistically significant (P<0.05) increase in absolute liver weight was observed when compared with the vehicle control. However, statistically significant (P<0.05) decreases were observed in the absolute liver weight when comparing the polycan 62.5 and 125 mg/kg groups with the vehicle control, with a non-significant decrease in the 31.25 mg/kg group. In addition, the differences between the relative liver weights in all the polycan groups and the vehicle control group were non-significant, but were shown to dose-dependently decrease (Table IV).

In the vehicle control group, the absolute liver weight was shown to change by 74.15% when compared with the sham group. In the SIMVA, polycan 31.25, 62.5 and 125 mg/kg groups, the absolute liver weight changed by 1.12, −16.49, −22.98 and −24.71% when compared with vehicle control group, respectively. In the vehicle control group, the relative liver weight was found to increase by 50.37% when compared with the sham group. In addition, the relative liver weights in the SIMVA, polycan 31.25, 62.5 and 125 mg/kg groups were found to change by 28.20, −9.53, −18.57 and −19.25% when compared with vehicle control group, respectively.

### Changes in the serum levels of AST and ALT

Statistically significant (P<0.01) increases in the serum levels of AST and ALT were detected when comparing the vehicle control group with the sham group, who were supplied a normal diet. In the SIMVA group, non-significant increases in the serum levels of AST and ALT were detected when compared with the vehicle control. However, statistically significant (P<0.01 or P<0.05) and dose-dependent decreases in the serum AST and ALT levels were demonstrated in all the polycan treatment groups when compared with the vehicle control group (Table V).

In the vehicle control group, the serum AST level was shown to increase by 218.53% when comparing with the sham group. In addition, the AST levels in the SIMVA, polycan 31.25, 62.5 and 125 mg/kg groups were demonstrated to change by 14.91, −44.36, −55.64 and −56.85% when compared with vehicle control group, respectively. Furthermore, in the vehicle control group, the serum ALT level was shown to increase by 170.92% of that observed in the sham group. In the SIMVA, polycan 31.25, 62.5 and 125 mg/kg groups, the changes in the ALT levels were 8.69, −34.02, −39.93 and −41.98% when compared with the vehicle control group, respectively.

### Changes in the serum levels of triglyceride and T-CHOL

Statistically significant (P<0.01) increases in the levels of serum triglyceride and T-CHOL were detected when comparing the vehicle control group with the normal diet supplied sham group. However, the serum triglyceride and T-CHOL levels in all the test article administration groups were significantly (P<0.01 or P<0.05) decreased when compared with the vehicle control. In the polycan groups, evident dose-dependent decreases were observed (Table V).

In the vehicle control group, the serum triglyceride level was found to increase by 175.76% when compared with the sham group. The triglyceride levels in the SIMVA, polycan 31.25, 62.5 and 125 mg/kg groups were demonstrated to change by −58.24, −39.01, −51.01 and −55.68% when compared with the vehicle control group, respectively. Furthermore, in the vehicle control group, the serum T-CHOL levels were shown to increase by 171.43% when comparing with the sham group. In the SIMVA, polycan 31.25, 62.5 and 125 mg/kg groups, the changes in the T-CHOL levels were −48.55, −25.65, −32.32 and −37.44% when compared with the vehicle control group, respectively.

### Changes in the serum levels of LDL and HDL

Statistically significant (P<0.01 or P<0.05) increases in the serum levels of LDL and HDL were detected in the vehicle control group when compared with the sham group, who were supplied with a normal diet. However, the serum LDL levels in all the test article administration groups were found to significantly (P<0.01) decrease when compared with the vehicle control group. No statistically significant differences were identified with regard to the serum HDL levels in all the test article administration groups when compared with the vehicle control group. In the polycan groups, an evident dose-dependent decrease in the serum LDL levels was observed (Table V).

In the vehicle control group, the serum LDL levels were found to be 348.39% of those in the sham group. Furthermore, the LDL levels in the SIMVA, polycan 31.25, 62.5 and 125 mg/kg groups were shown to change by −47.48, −19.66, −23.02 and −24.46% when compared with the vehicle control group, respectively. In the vehicle control group, the serum HDL levels increased by 71.75% when compared with the sham group. In the SIMVA, polycan 31.25, 62.5 and 125 mg/kg groups, the HDL levels were shown to change by 9.17, 1.45, 3.95 and 8.52% when compared with the vehicle control group, respectively.

### Changes in the histopathology and histomorphometry of the liver

**Figure 1:**
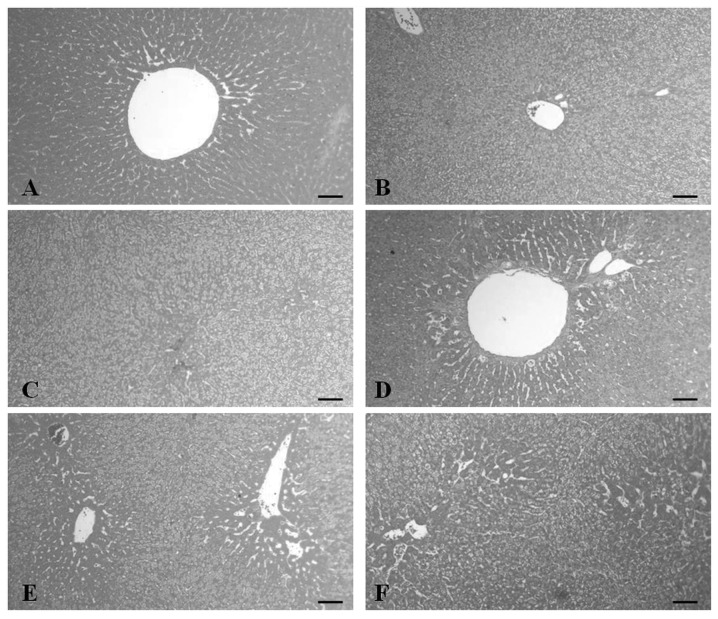
Changes to the histological profiles of the liver in the (A) sham, (B) vehicle control, (C) simvastatin (SIMVA), (D) polycan 31.25 mg/kg, (E) polycan 62.5 mg/kg and (F) polycan 125 mg/kg groups at sacrifice. Only slight fatty changes were detected in the sham group; however, severe fatty changes were detected throughout all the hepatic lobules in the vehicle control and SIMVA groups. A marked, dose-dependent decrease in fatty changes was detected in the polycan groups. All the images were stained with hematoxylin and eosin (scale bars, 100 μm).

Fatty changes were detected throughout all the hepatic lobules in all groups (Fig. 1) and similar fatty changes were detected in all the treatment groups. In addition, the percentage of fatty changes in the hepatic regions significantly (P<0.01) increased in the vehicle control group when compared with the sham group. The degenerative regions in the SIMVA group were quite similar to those in the vehicle control group. However, statistically significant (P<0.01) and dose-dependent decreases were observed in the percentage of regions exhibiting fatty changes in the liver parenchyma when comparing all the polycan groups with the vehicle control group (Table VI). In addition, the severity of liver steatosis markedly and dose-dependently decreased in all the polycan groups when compared with the vehicle control group (Fig. 1).

In the vehicle control group, the percentage of fatty change hepatic regions was shown to be 10,874.07% of those observed in the sham group. In the SIMVA, polycan 31.25, 62.5 and 125 mg/kg groups, the percentage of degenerative regions was shown to change by 0.71, −9.77 −17.39 and −18.45% when compared with the vehicle control group, respectively.

### Changes in the histopathology and histomorphometry of the aorta

**Figure 2:**
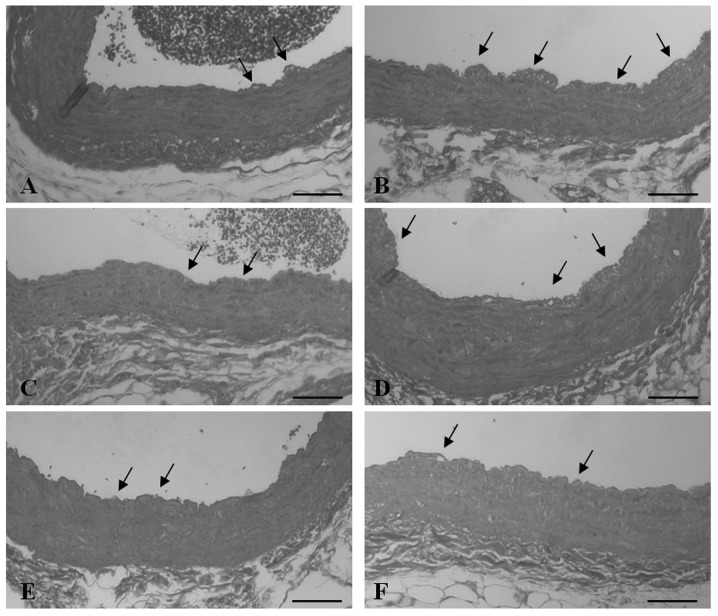
Changes to the histological profiles of the abdominal aorta in the (A) sham, (B) vehicle control, (C) simvastatin (SIMVA), (D) polycan 31.25 mg/kg, (E) polycan 62.5 mg/kg and (F) polycan 125 mg/kg groups at sacrifice. Small atherosclerotic plaques, consisting of foam cells, were detected in the sham group at a low frequency; however, numerous and relatively broad atherosclerotic plaques were detected in the tunica intima of the vehicle control group. A marked and dose-dependent decrease was observed in the polycan groups, and in the SIMVA group, when compared with the vehicle control. Arrows indicate the atherosclerotic plaques. All the images were stained with hematoxylin and eosin (scale bars, 100 μm).

**Figure 3:**
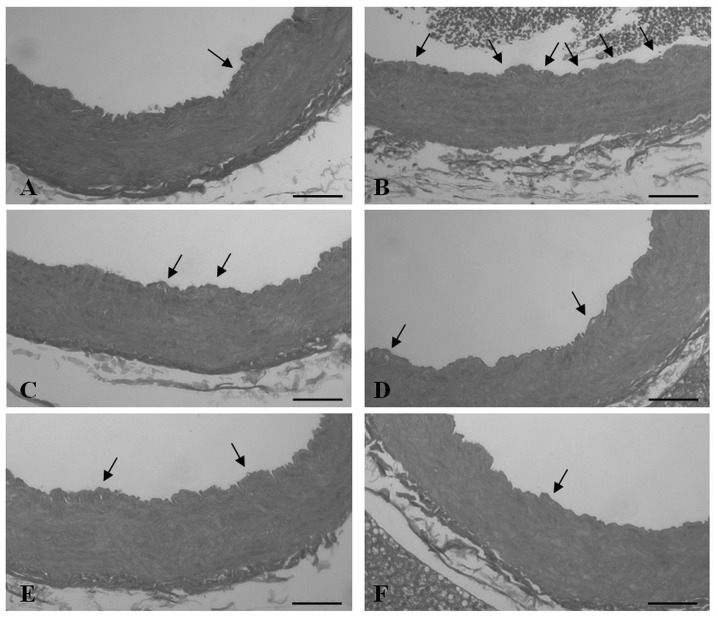
Changes to the histological profiles of the thoracic aorta in the (A) sham, (B) vehicle control, (C) simvastatin (SIMVA), (D) polycan 31.25 mg/kg, (E) polycan 62.5 mg/kg and (F) polycan 125 mg/kg groups at sacrifice. Small atherosclerotic plaques, consisting of foam cells, were detected in the sham group at a low frequency; however, numerous and relatively broad atherosclerotic plaques were detected in the tunica intima of the vehicle control group. A marked and dose-dependent decrease in the number of plaques was observed in the polycan groups, and in the SIMVA group, when compared with the vehicle control. Arrows indicate the atherosclerotic plaques. All the images were stained with hematoxylin and eosin (scale bars, 100 μm).

Atherosclerotic plaques consisting of foam cells were detected throughout the whole aortic surface of the vehicle control. However, the incidence of these atherosclerotic plaques in all the treatment groups was shown to markedly decrease compared with the vehicle control group, regardless of whether the aorta was thoracic or abdominal (Figs. 2 and 3). In addition, the percentage of atherosclerotic plaques on the aorta surface was significantly (P<0.01) increased in the vehicle control group when compared with the sham group. However, the percentage of atherosclerotic plaques significantly (P<0.01) decreased in all the treatment groups when compared with the vehicle control group. In the polycan groups, an evident dose-dependent decrease was observed in the percentage of atherosclerotic plaques (Table VI).

In the vehicle control group, the percentage of atherosclerotic plaques in the thoracic aorta was shown to be 1,826.85% greater when compared with the sham group. In the SIMVA, polycan 31.25, 62.5 and 125 mg/kg groups, the percentage of atherosclerotic plaques in the thoracic aorta was shown to change by −87.77, −66.00, −74.60 and −80.80% when compared with the vehicle control group, respectively.

In the vehicle control group, the percentage of atherosclerotic plaques in the abdominal aorta was shown to increase by 1,164.80% when compared with the sham group. Furthermore, in the SIMVA, polycan 31.25, 62.5 and 125 mg/kg groups, the percentage of plaques were shown to change by −80.96, −44.04, −66.70 and −70.42% when compared with the vehicle control group, respectively.

## Discussion

Hypolipemic and associated anti-atherosclerosis effects of β-glucan have been previously investigated (16,17), with evidence demonstrating the favorable effects of β-glucan on the hepatopathies (18,19). However, the direct effects of β-glucan on hyperlipemic liver damage, and the effects on hypolipemia and associated anti-atherosclerosis using the β-glucan originating from *Aureobasidium*, have not yet been determined. In the present study, the β-glucan used was extracted from *A. pullulans* SM-2001 (mainly β-1,3/1,6-glucans), a UV-induced mutant of *A. pullulans* that demonstrates different characteristics compared with other types of β-glucan, derived from various origins (20). In the present study, the hypolipemic and associated anti-atherosclerosis effects of the test articles were observed on a HFD-induced hyperlipemia hamster model, with regard to assessing the possible effects on liver damage. The effects were evaluated based on the serum levels of AST, ALT, LDL, HDL, T-CHOL and triglyceride, with changes to histology and histomorphometry in the liver and aorta (thoracic and abdominal) also observed. As a result of the HFD supply, statistically significant (P<0.01 or P<0.05) decreases were observed in food consumption, while hyperlipemia and associated liver steatosis (damages) markedly increased. In addition, increases were observed in the body weight, liver weight and serum levels of AST, ALT, LDL, HDL, triglyceride and T-CHOL in all the HFD supplied groups when compared with the sham group who were fed a normal pellet diet. Furthermore, severe fatty changes and atherosclerotic plaques were observed on the liver and aorta surface, respectively, with the percentage of fatty change regions in the liver and the percentages of atherosclerotic plaques increasing significantly. However, the effects on hyperlipemia were shown to markedly and dose-dependently decrease in all the test article administration groups, with the exception of the HDL level in the SIMVA group. No statistically significant differences were observed in the HDL levels. More severe hepatic damage was detected in the SIMVA group when compared with the vehicle control group, with the SIMVA group showing significantly increased serum AST and ALT levels. However, statistically significant and dose-dependent decreases were observed with regard to the hyperlipemia-associated liver damage in the polycan treatment groups, with changes in the histology and serum biochemistry compared with the vehicle control group. Based on the results of the present study, polycan was demonstrated to exert favorable effects with regard to decreasing HFD-induced hyperlipemia and associated atherosclerosis, with relatively good protective effects on liver damage.

An increase in body weight following hyperlipemia is generally observed (27), and this can be used as a type of animal model in the development of antiobesity agents (28). Similar to the observations of previous studies (27,29), a significant increase was detected in the body weight of the HFD supplied groups when compared with the sham group in the present study. However, no statistically significant differences were observed when comparing the body weights of the hamsters in the polycan groups with the vehicle control group. The change detected in the SIMVA group was considered to be a secondary effect caused by hepatotoxicity since more substantial hepatic damage was detected in this group compared with the vehicle control.

A decrease in food consumption was detected in all the HFD supplied groups, which was considered to be the result of the HFD and the time schedule of the study. In the present study, to evaluate the preventative effects of the study treatments, the acclimatization period to HFD was excluded. However, food consumption did not differ among the HFD supplied groups, and the body weight was not found to decrease compared with that in the normal diet supplied sham group, as previously discussed (17).

In general, liver damage is accompanied with hyperlipemia (30), and changes to the liver weight and the serum levels of AST and ALT function as serum markers of liver damage, which are generally monitored in HFD-induced hyperlipemia. Although a number of materials exhibiting hypolipemic effects have been reported to show hepatoprotective effects, particularly in herbal extracts (30–32), SIMVA has been demonstrated to increase the serum levels of AST and ALT (33). In the present study, although similar histological profiles of the liver and histomorphometrical changes to the fatty change regions were observed, an increase in the serum levels of AST and ALT were detected in the SIMVA group, with an increase in liver weight also. Thus, SIMVA was considered to aggravate the hepatic damage induced by the HFD supply. However, the hyperlipemia-associated hepatic damages were significantly and dose-dependently decreased in all the polycan treatment groups, with changes observed in the liver weight, serum AST and ALT levels, and histology and histomorphometry of the liver parenchyma. Therefore, polycan was considered to exert a number of favorable effects in preventing hyperlipemia-associated hepatic damage. Previous studies have revealed that certain antioxidants may scavenge free radicals and inhibit lipid peroxidation; thus, treatment with several antioxidants has been shown to protect against free radical-induced hepatic damage (34,35). In addition to chemically synthesized antioxidants, dietary antioxidants have also been shown to protect against CCl_4_-induced lipid peroxidation (36). Therefore, the mechanisms underlying the hepatoprotective effects detected in the present study were considered to be associated with the antioxidative and free radical scavenging activities of β-glucan (37,38).

Generally, the most critical problem in hyperlipemia is the increase in the serum levels of LDL, triglyceride and T-CHOL, with a decrease in the HDL level (39–41). The efficacy of hypolipemic agents is generally evaluated based on the decrease in serum LDL, triglyceride and T-CHOL levels, and the increase in the HDL level (42–44). In the present study, the serum HDL levels in the vehicle control group were shown to increase compared with the sham group, which differs to previous studies (3,39,43). These differences are considered to be the result of using an animal model, as shown previously with the use of animals (45). In the present study, marked and dose-dependent decreases were observed in the serum levels of LDL, triglyceride and T-CHOL, which demonstrates the evident hypolipemic effects of polycan. No statistically significant differences were detected in the serum HDL levels compared with the vehicle control, similar to the observations of the SIMVA group.

In addition, atherosclerotic plaques, consisting of foam cells, have been previously detected on the aortic surface of hyperlipemic animals (5), and these atherosclerotic plaques have been used as index for determining anti-atherosclerosis effects (46,47). In the present study, the incidence of these atherosclerotic plaques in the polycan and SIMVA treatment groups were shown to markedly and dose-dependently decrease when compared with the vehicle control group, regardless of whether the plaques were identified in the thoracic or abdominal aorta. In addition, the percentage of atherosclerotic plaques on the aorta surfaces was shown to significantly decrease in the treatment groups when compared with the vehicle control group. Thus, the present study provides direct evidence that polycan exerts a number of anti-atherosclerosis effects following HFD induction.

A number of mechanisms are hypothesized to contribute to the ability of soluble fibers and their specific components to lower the serum cholesterol levels. Previous studies have demonstrated that the consumption of β-glucan inhibits the absorption of cholesterol from the gut, as demonstrated by a significant increase in the excretion of fecal cholesterol and neutral sterols (48–50). Thus, the cholesterol-lowering properties of β-glucan, at least in part, are considered to be the result of the inhibition of cholesterol absorption from the gut. In addition, fibers containing β-glucan have been reported to increase the excretion of bile acids, indicating a causative role in the decrease of the plasma cholesterol concentration (49,51,52). Therefore, similar concentrations of bile acid, relative to the fecal weight, may represent an increase in the bulk excretion of bile acids. However, the present study did not evaluate fecal output.

In conclusion, the results of the present study demonstrated that polycan exerts favorable effects in decreasing the extent of HFD-induced hyperlipemia and associated atherosclerosis. In addition, polycan was shown to exert relatively good protective effects on liver damage.

## Figures and Tables

**Table I:** Composition of the high-fat diet used in the study.

Ingredient	kcal/gm	g/kg	kcal/kg
Casen	3.72	200	744
DL-methionine	4	3	12
Cornstarch	3.6	150	540
Sucrose	4	487.5	1950
Cellulose	0	50	0
Corn oil	9	50	450
Mineral mix	0.47	35	16.45
Vitamin mix	3.92	10	39.2
Choline bitartrate	0	2	0
Sodium cholate	0	2.5	0
Cholesterol	0	10	0

High-fat diet was purchased from Dyets, Inc. (Bethlehem, PA, USA). Kcal/gm, calories per gram material.

**Table II:** Changes to the body weight (g) in the HFD-induced hyperlipemic hamster model.

				Polycan groups (mg/kg)		
				
Time point	Sham	Control	SIMVA	31.25	62.5	125
Day -1	97.62±12.08	95.44±4.43	96.40±8.75	95.82±2.06	95.32±8.73	96.66±12.72
Day 0^a^	93.48±12.33	92.20±4.19	91.36±8.24	92.30±2.92	90.24±8.33	87.68±17.28
Day 1	96.00±13.34	90.54±4.34	90.46±8.20	90.34±2.30	86.98±7.25	89.60±14.36
Day 7	100.86±14.69	90.24±12.58	95.66±8.21	92.00±5.19	84.22±10.08	94.86±15.28
Day 14	103.76±7.64	94.90±12.09	95.24±5.45	96.24±7.90	91.62±11.15	100.32±15.93
Day 21	107.10±8.00	100.26±13.92	101.86±6.87	101.60±11.55	96.64±18.65	105.02±16.48
Day 28	118.30±9.63	110.92±14.70	103.08±11.45^d^	110.24±16.12	106.78±15.91	112.44±15.78
Day 35	123.34±9.10	116.28±13.41	106.02±15.58^d^	119.46±19.53	112.88±17.21	124.18±9.85
Day 42	131.58±7.41	133.64±14.08	117.96±16.38	130.76±13.34	126.84±9.26	129.98±8.82
Day 49	133.00±6.94	145.06±9.66^d^	129.64±20.84	139.74±20.31	144.90±13.18	138.48±7.75
Day 55	134.30±7.56	158.42±12.57^d^	126.38±25.79	148.10±21.14	152.16±19.10	149.56±16.77
Day 56^b^	126.70±6.87	147.58±10.93^d^	116.80±24.84	136.70±16.41	140.38±17.49	138.14±15.70
Gain^c^	33.22±10.97	55.38±8.95^d^	25.44±18.38^e^	44.40±14.60	50.14±15.42	50.46±27.13

Results are expressed as the mean ± standard deviation (n=5).

a Initial dose of the test article and HFD supply after overnight fasting;

b At sacrifice after overnight fasting;

c Body weight gain (g) = body weight gain throughout the whole experimental period (day 0 - day 56).

d P<0.05, vs. sham (MW test);

e P<0.05, vs. vehicle control (MW test).

SIMVA, simvastatin; HFD, high-fat diet; MW, Mann-Whitney-Wilcoxon.

**Table III:** Changes in the food consumption (g) in the high-fat diet-induced hyperlipemic hamster model.

Time points	Sham	Control	SIMVA	Polycan groups (mg/kg)		

				31.25	62.5	125
Day 1	6.74	5.12	4.98	5.10	5.36	5.58
Day 7	6.10	4.04	5.94	5.34	5.76	5.08
Day 14	5.94	4.64	5.28	5.34	4.54	5.70
Day 21	6.02	5.66	5.34	6.12	4.30	4.30
Day 28	8.14	4.26	4.54	5.72	4.44	4.92
Day 35	8.44	6.30	4.25	5.20	5.20	5.78
Day 42	8.93	5.66	5.71	4.80	5.90	6.43
Day 49	7.22	5.80	6.22	4.22	5.80	5.75
Day 55	7.12	6.23	5.89	4.33	5.72	5.16
Mean^a^	7.18±1.11	5.30±0.83^b^	5.35±0.66^b^	5.13±0.61^b^	5.22±0.64^b^	5.41±0.62^b^

a Results are expressed as the mean ± standard deviation (n=5).

b P<0.01, vs. sham (MW test).

SIMVA, simvastatin; MW, Mann-Whitney-Wilcoxon.

**Table IV:** Changes in the absolute and relative liver weights in the high-fat diet-induced hyperlipemic hamster model.

Group	Absolute weight (g)	Relative weight (%)
Sham	3.682±0.405	2.907±0.277
Control	6.413±1.063^a^	4.372±0.876^a^
SIMVA	6.485±1.137^a^	5.605±0.562^a^,^c^
Polycan 31.25	5.356±0.797^a^	3.955±0.745^b^
Polycan 62.5	4.939±0.571^a^,^c^	3.560±0.582^a^
Polycan 125	4.828±0.427^b^,^c^	3.530±0.479^b^

Results are expressed as the mean ± standard deviation (n=5). Relative liver weight (%) was calculated as follows: (Absolute liver weight/body weight at sacrifice) × 100.

a P<0.01

b P<0.05, vs. sham (MW test);

c P<0.05, vs. vehicle control (MW test).

SIMVA, simvastatin; MW, Mann-Whitney-Wilcoxon.

**Table V:** Changes in the serum biochemistry of the high-fat diet-induced hyperlipemic hamster model.

Group	AST (IU/l)	ALT (IU/l)	Triglyceride (mg/dl)	T-CHOL (mg/dl)	LDL (mg/dl)	HDL (mg/dl)
Sham	51.80±5.01	61.20±7.40	79.20±20.89	99.40±17.05	18.60±0.89	65.76±15.47
Control	165.00±15.28^a^	165.80±38.92^a^	218.40±38.00^a^	269.80±40.06^a^	83.40±5.18^a^	112.94±19.84^b^
SIMVA	189.60±26.00^a^	180.20±24.02^a^	91.20±20.02^b^,^c^	138.80±38.15^c^	43.80±10.33^a^,^c^	123.30±38.77^b^
Polycan 31.25	91.80±34.59^a^,^c^	109.40±37.00^a^,^d^	133.20±30.81^b^,^c^	200.60±43.19^a^,^d^	67.00±12.92^a^,^d^	114.58±14.38^a^
Polycan 62.5	73.20±7.89^a^,^c^	99.60±9.37^a^,^c^	107.00±35.55^c^	182.60±32.85^a^,^d^	64.20±5.63^a^,^c^	117.40±18.85^b^
Polycan 125	71.20±24.35^a^,^c^	96.20±8.58^a^,^c^	96.80±26.45^c^	168.80±53.44^b^,^c^	63.00±9.57^a^,^c^	122.56±18.97^a^

Results are expressed as the mean ± standard deviation (n=5).

a P<0.01 and

b P<0.05, vs. sham (MW test);

c P<0.01 and

d P<0.05, vs. vehicle control (MW test).

SIMVA, simvastatin; AST, aspartate aminotransferase; ALT, alanine aminotransferase; T-CHOL, total cholesterol; LDL, low-density lipoprotein; HDL, high-denisty lipoprotein; MW, Mann-Whitney-Wilcoxon.

**Table VI:** Changes to the histomorphometry of the liver and aorta in the high-fat diet-induced hyperlipemic hamster model.

Group	Fatty change regions (%/200 μm^2^ liver parenchyma)	Atherosclerotic plaques (%/1 mm aorta surface)	

		Abdominal aorta	Thoracic aorta
Sham	0.86±0.39	0.64±0.56	0.43±0.17
Control	93.94±2.88^a^	8.12±1.89^a^	8.32±2.33^a^
SIMVA	94.61±2.73^a^	1.55±0.48^b^,^c^	1.02±0.19^a^,^c^
Polycan 31.25	84.76±4.93^a^,^c^	4.54±1.20^a^,^c^	2.83±0.87^a^,^c^
Polycan 62.5	77.60±8.62^a^,^c^	2.70±1.13^a^,^c^	2.11±0.69^a^,^c^
Polycan 125	76.61±8.74^a^,^c^	2.40±1.36^b^,^c^	1.60±0.93^a^,^c^

Results are expressed as the mean ± standard deviation (n=5).

a P<0.01 and

b P<0.05, vs. sham (MW test);

c P<0.01, vs. vehicle control (MW test).

SIMVA, simvastatin; MW, Mann-Whitney-Wilcoxon.
